# Pancreatic carcinoma masked as fever of unknown origin: A case report and comprehensive review of literature

**DOI:** 10.1097/MD.0000000000004643

**Published:** 2016-09-02

**Authors:** Ning Shi, Cheng Xing, Xiaoyan Chang, Menghua Dai, Yupei Zhao

**Affiliations:** a Department of General Surgery; b Department of Pathology, Peking Union Medical College Hospital, Chinese Academy of Medical Sciences and Peking Union Medical College, Beijing, China.

**Keywords:** fever of unknown origin, neoplastic fever, pancreatic carcinoma

## Abstract

**Background:**

Pancreatic carcinoma is a highly lethal malignancy. Common presenting features of pancreatic cancer include anorexia, asthenia, weight loss, pain, and obstructive jaundice. Nevertheless, fever as a symptom, or even primary manifestation of pancreatic cancer is rather rare.

**Methods:**

Here, we report a 63-year-old male patient presenting with daily fevers, night sweats, and fatigue of 2-month duration. Laboratory findings showed elevated white blood cell count (WBC), erythrocyte sedimentation rate (ESR), and serum C-reactive protein (CRP). A computed tomography scan demonstrated a tumor between the duodenum and pancreatic head. Chest radiograph was normal.

**Results:**

The patient underwent an uneventful tumor resection. Histological examination of a surgical specimen demonstrated an undifferentiated adenocarcinoma originated from pancreatic head. The tumor was compatible with TNM stage IIA (T3N0M0). Complete resolution of the fever was achieved on post-operative day 4 and no recurrence of the tumor or neoplastic fever happen during the 39-month follow-up.

**Conclusion:**

Pancreatic adenocarcinoma could manifest as neoplastic fever at the time of diagnosis. If the tumor is resectable, surgical resection is a safe and curative form of therapy not only for the fever but also for the original carcinoma.

## 1 Introduction

Pancreatic carcinoma is a highly lethal malignancy and over 90% of pancreatic cancers are ductal adenocarcinomas of the exocrine pancreas. These tumors occur twice as frequently in the pancreatic head compared with the rest of the organ.^[1]^ Common presenting features of pancreatic cancer include anorexia, asthenia, weight loss, pain, and obstructive jaundice. Patients with jaundice tend to be diagnosed at an earlier stage of the disease.^[2]^ Other symptoms tend to be more insidious; therefore, in the absence of jaundice, the interval between onset and diagnosis can be prolonged.^[3]^ Nevertheless, fever as a symptom, or even primary manifestation of pancreatic cancer is rather rare.

Infection is the most frequent (67%) etiology of fever in cancer patients, while neoplastic fever, which is caused by the tumor itself or its invasive procedure, accounts for 27% of the non-infectious febrile episodes.^[4]^ Some different tumors that initially manifest themselves as neoplastic fever have been reported, such as hematological malignancies, colon cancer, renal cell carcinoma, and cholangiocarcinoma.^[5–8]^ However, pancreatic cancer is scarcely associated with neoplastic fever.

The diagnosis of neoplastic fever can be made only after excluding identifiable etiologies; therefore, fever in cancer patients usually poses a diagnostic dilemma. Herein, we describe an unusual case of nonmetastatic pancreatic carcinoma, which primarily manifested as fever of unknown origin (FUO). The ethics committee of Peking Union Medical College Hospital approved of this study.

## 2 Case report

A 63-year-old man was referred to our hospital presenting daily fevers, night sweats, anorexia, and fatigue for the previous 2 months along with weight loss of 5 kg. He denied chills, abdominal pain, diarrhea, and arthralgias. A detailed physical examination revealed a diaphoretic, well-nourished man with an elevated temperature (38 °C) but no sign of jaundice or rash. The patient had a history of left Achilles tendon rupture and underwent a curative operation 47 years ago, and a history of smoking for 45 years, consuming 6 cigarettes per day averagely.

An extensive outpatient and inpatient diagnostic workup was initiated. The white blood cell count (WBC) was elevated (13 × 10^9^/L), and urinalysis, routine stool, and stool occult blood test were negative. The levels of serum C-reactive protein (CRP) and erythrocyte sedimentation rate (ESR) were remarkably elevated (173 mg/L and 118 mm/h, respectively), but the level of serum procalcitonin was normal (0.11 ng/mL). Repeated cultures of blood and sputum were negative, and serologies for mycoplasma pneumonia, chlamydia pneumonia, brucellosis, and tuberculosis were unrevealed. Further laboratory examinations including anti-neutrophil cytoplasmic antibodies, anti-nuclear antibodies, and anti-extractable nuclear antigen antibodies were negative. Chest radiograph was normal.

**Figure 1:**
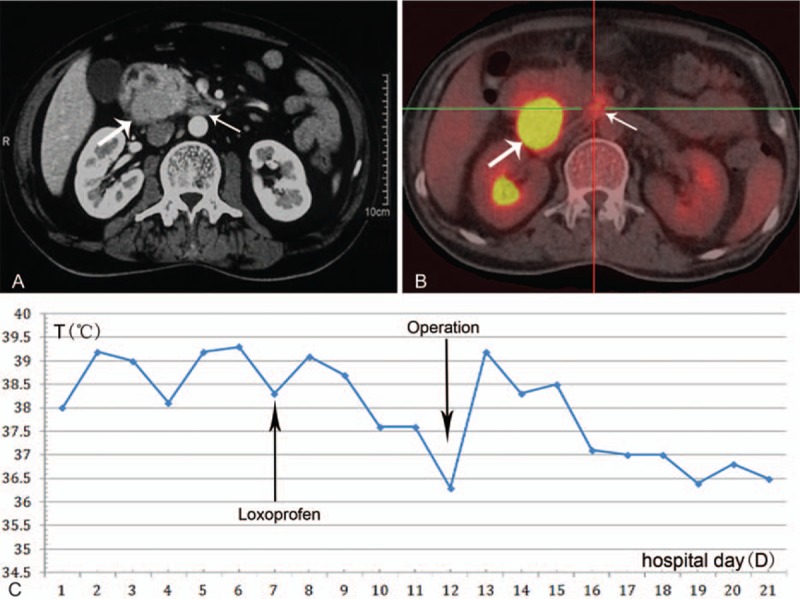
(A) Abdominal CT scan revealed a tumor between the duodenum and the pancreatic head (bold arrow) and enlarged retroperitoneal lymph nodes (thin arrow). (B) PET-CT revealed the mass (bold arrow) and enlarged lymph nodes (thin arrow) to have significantly elevated standard uptake value (SUV). (C) Temperature decreased to below 37.6 °C for 2 days before the surgery due to the therapy with loxoprofen, and complete resolution occurred on postoperative day 4. CT = computed tomography, PET-CT = positron emission tomography-computed tomography.

Abdominal ultrasonography indicated multiple gallstones and a mass on the right side of superior mesenteric vein. A computed tomography scan revealed a tumor (5.5 × 3.3 × 3.2 cm) between the duodenum and pancreatic head with visibly enlarged retroperitoneal lymph nodes (Fig. 1A). Positron emission tomography-computed tomography (PET-CT) demonstrated that the mass and swollen lymph nodes had significantly elevated standard uptake value (SUV), indicating a malignant lesion accompanied with lymphatic-metastasis (Fig. 1B). Biopsy samples obtained with endoscopic ultrasonography indicated malignant cells, which further supported the diagnosis of poorly differentiated adenocarcinoma based on the results of immunohistochemical staining: CA125 (+), CA199 (−), CD56 (−), CDX2 (−), CEA (−), CK19 (+), CK7 (+), CgA (−), Ki-67 (+, 10%), Syn (−), Vim (−), and β-catenin (+).

The case was discussed at a multidisciplinary team meeting. Empirical antibiotics were administrated for over 7 days for suspected sepsis, but failed to elicit a response. The tumor in the pancreaticoduodenal region was the likely cause of the fever. To alleviate morbidity resulting from the fever, the patient was treated with loxoprofen 30 mg q.d. for 5 days before operation (Fig. 1C). The body temperature decreased to below 37.6 °C for 2 days. Then, pancreaticoduodenectomy and lymphadenectomy were performed on this patient. Loxoprofen administration was stopped immediately after the surgical resection.

**Figure 2:**
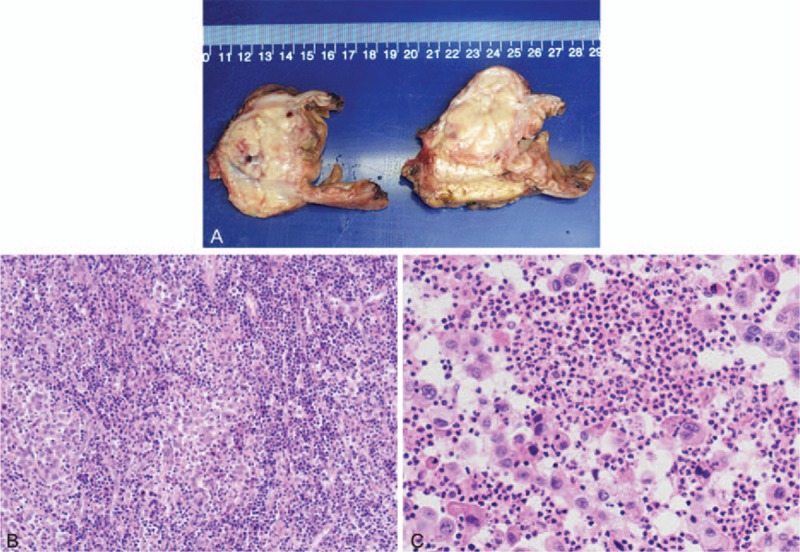
(A) Pathological examination of the resected specimen revealed a mass (4.5 × 2.9 × 4.0 cm) originating from pancreatic head and was later diagnosed as undifferentiated adenocarcinoma. (B) A large number of lymphocytes infiltrated the tumor diffusely (Hematoxylin and eosin, 100 times). (C) Infiltration of polymorphonuclear leukocytes (PMNs) were visible within the tumor (Hematoxylin and eosin, 400 times).

Interestingly, complete resolution of the fever was achieved on postoperative day 4 (Fig. 1C), and the WBC normalized on postoperative day 5. This strongly corroborates a diagnosis of neoplastic fever. Histopathologic examination of the resected specimen revealed a mass (4.5 × 2.9 × 4.0 cm) originating from the pancreatic head (Fig. 2A), which was diagnosed as undifferentiated adenocarcinoma involving the duodenum and peripancreatic tissue. A large number of lymphocytes infiltrate the tumor diffusely (Fig. 2B) and infiltration of multiple clusters of polymorphonuclear leukocytes (PMNs) was visible within the tumor (Fig. 2C). All margins of the tumor including the retroperitoneal margin were negative. Thirty-three resected lymph nodes were not involved but some were categorized as reactive hyperplasia. The tumor was compatible with TNM stage IIA (T3N0M0).

Subsequently, the patient received 6 courses of adjuvant chemotherapy with gemcitabine (1000 mg/m^2^, 30 min intravenous infusion, at day 1, 8, and 15, repeated every 28 days) from the day after postoperative day 30. There was no recurrence of the tumor or neoplastic fever during the 39-month follow-up.

## 3 Discussion

Classic FUO is defined as fever ≥38.3 °C (101 °F) for 3 or more weeks in immunocompetent, otherwise healthy patients with no identified cause of fever after undergoing a set of obligatory investigations.^[9]^ In 60% of such patients FUO is related to infection, while neoplastic fever accounts for 27% of those with non-infectious fever.^[10]^ Neoplastic fever, which has been defined as fever caused by cancer itself, is a well-described entity in patients with hematological malignancies, colon cancer, renal cell carcinoma.^[11]^ It is more frequently encountered in the metastatic setting, especially with metastasis to the liver.^[12,13]^ Here, we describe an unusual case of non-metastatic pancreatic carcinoma presenting as neoplastic fever.

The pathophysiological mechanism of neoplastic fever remains uncertain. One theory is that cancer cells or host cells in response to tumor can produce some pyrogenic cytokines including interleukin (IL)-1, IL-6, IL-8 tumor necrosis factor (TNF)-α, and interferon.^[14–16]^ These cytokines activate the anterior preoptic nuclei of the hypothalamus and raise the set point for body temperature through the induction of prostaglandin E2.^[15]^ The pathological examination of our patient showed an undifferentiated carcinoma infiltrated by a large number of lymphocytes and multiple clusters of PMNs. The enlarged draining lymph nodes were diagnosed as reactive hyperplasia. These dramatically increased lymphocytes and PMNs may result in neoplastic fever by releasing enormous pyrogenic cytokines. Other mechanisms for neoplastic fever include tumor necrosis, release of TNF, and other pyrogens from dead tissue.^[15]^ The pathological examination revealed no evidence of necrosis within the tumor of our patient. Neoplastic fever in patients with brain metastases is suspected to be one example of neurogenic fever,^[17]^ which has been attributed to direct brain tissue damage and subsequent activation of phospholipase A2.^[18]^ However, no distant metastases had been found in our patient.

Since administrating appropriate antibiotics timely can impact the outcome of patients with specific infections significantly, it is crucial to differentiate between infectious fever and neoplastic fever.^[19,20]^ Typically, fever due to infection is associated with chills, warmth, and periodic sweating and is sometimes accompanied with tachycardia, hypotension, and mental status changes, particularly in gram-negative bacteremia. However, neoplastic fever is usually characterized by a sensation of warmth and sweating but less frequently by chills, tachycardia, and mental status changes.^[21]^ With regard to laboratory tests, CRP and ESR levels at admission are not clinically useful in differentiation between neoplastic and infectious fever.^[22]^ Yaegashi et al^[23]^ found that the procalcitonin level was significantly higher in patients with bacterial infections than patients with neoplastic fever caused by urological cancer. The naproxen test is a classic approach to distinguish neoplastic from non-neoplastic fever.^[21]^ On a naproxen test, naproxen is administered, and reactions are regarded as positive if pyrexia diminishes 24 hours after administration. Study by Tsavaris et al^[24]^ reported that the sensitivity and specificity of this test were 92% and 100%, respectively. Nevertheless, study from Vanderschueren demonstrated conflicting results.^[25]^

When the diagnosis of neoplastic fever is established, disease-specific therapy such as operation, radiotherapy, or chemotherapy may be useful to palliate the fever.^[26]^ In the study by Chawla et al,^[27]^ 7 of 17 (41%) patients with metastatic breast cancer responded to systematic anti-neoplastic therapy and neoplastic fever lysed only in this group of responders. Additionally, non-steroidal anti-inflammatory drugs have been reported with significant efficacy in treating neoplastic fever, including naproxen, diclofenac, indomethacin, rofecoxib, and ibuprofen.^[24,28]^ The fever of our patient responded well to administration of loxoprofen, reinforcing the diagnosis of neoplastic fever. Nevertheless, possible gastrointestinal side effects of non-steroidal anti-inflammatory drugs should also be considered along with the potential benefits. Furthermore, neoplastic fever will recur in some patients if NASID is discontinued after a short-term treatment.^[29]^

Undifferentiated carcinoma is one of variants of pancreatic ductal adenocarcinoma. It is divided into the following four categories: anaplastic giant cell carcinoma, sarcomatoid carcinoma, carcinosarcoma, and undifferentiated carcinoma with osteoclast-like giant cells. In contrast to typical ductal adenocarcinoma, undifferentiated carcinomas are poorly cohesive, cellular, and often have only scant amount of stroma.^[30,31]^ Several studies have reported that the prognosis of undifferentiated carcinoma is extremely poor, and average survival is as short as 5 months.^[30,32,33]^ However, the patient with pancreatic undifferentiated carcinoma in the present report had a favorable prognosis (has not shown evident recurrence for 39 months). This may result from the acute manifestation of fever leading to earlier diagnosis and enormous lymphocytes and PMNs infiltration impeding the progression of the tumor.

## 4 Conclusion

Finally, we conclude that pancreatic adenocarcinoma can manifest as neoplastic fever at the time of diagnosis, and if the tumor is resectable, surgical resection is a safe and curative form of therapy not only for the fever but also for the original carcinoma.
